# Development and Application of an In-house Line Probe Assay for Hepatitis C Virus Genotyping

**DOI:** 10.5812/hepatmon.6767

**Published:** 2013-04-30

**Authors:** Keivan Majidzadeh-A, Abbas Morovvati, Mohammad Soleimani, Arash Ghalianchi Langeroudi, Shahin Merat, Hossain Jabbari

**Affiliations:** 1 Tasnim Biotechnology Research Center (TBRC), Faculty of Medicine, AJA University of Medical Sciences, Tehran, IR Iran; 2 Academic Center for Culture, Education & Research (ACECR), Iranian Center for Breast Cancer (ICBC), Tehran, IR Iran; 3 Department of Microbiology, Faculty of Veterinary Medicine, University of Tehran, Tehran, IR Iran; 4 Digestive Disease Research Center, Tehran University of Medical Sciences, Tehran, IR Iran

**Keywords:** Hepatitis C Virus, Genotyping, LIPA Protein, Human

## Abstract

**Background:**

Hepatitis C virus (HCV) is the major cause of chronic liver disease. HCV is a single stranded positive sense RNA of approximately 9.6 Kb. Because of high conservativeness of 5΄untranslated region of HCV genome, it is widely used for virus genotyping. Different methods are used for the virus genotyping, but all involve some difficulties.

**Objectives:**

The aim of the present study was to develop an in-house reverse hybridization method as a line probe assay, for HCV genotyping.

**Materials and Methods:**

Sixty serum samples were collected with newly diagnosis of HCV infection. Genotyping process had already been performed for the samples using RT-PCR RFLP method. After total RNA extraction from the samples and cDNA synthesis, nested PCR method was applied for amplification of the target sequence on the 5΄UTR. In the nested PCR, biotinylated oligonucleotides were used as inner primers. Optimized concentrations of the biotinylated inner primers (as positive control), two universal and seven specific probes were spotted onto nylon membrane stripes in a defined pattern. Hybridization process was conducted between the probes and the denaturized biotin labeled PCR products. Finally, the stripes were developed by using streptavidin conjugated alkaline phosphate as a signal generating agent. To determine the diagnostic sensitivity and specificity of the home made LiPA, a panel containing 60 confirmed sera with positive results for HCV (and PCR-RFLP genotyped) was subjected to evaluate.

**Results:**

Agarose gel electrophoresis of the nested PCR products using the outer and inner primers showed 305 and 234 bp fragments respectively. After performing hybridization and detection processes on the prepared strips, the colored bands were formed for the positive control, universal probes and the corresponding genotypes. HCV genotype results were found to be in 100% concordance through studying 60 sera that were successfully typed by the two methods. P-value of 0.045 conveys that the two methods were the same and had no significant difference.

**Conclusions:**

The most common genotyping method in Iran is RT-PCR RFLP. Given the results and advantages of this homemade technique, such as high specificity and sensitivity, ability for detection of most genotypes, it provides possibility of evaluating much of the isolates without needing electrophoresis stage.

## 1. Background

Hepatitis C virus is one of the major causes of the liver diseases. It is an approximately 9.6 kb single stranded positive sense RNA virus. This virus belongs to the Flaviviridae family and Hepacivirus genus. The structure of the RNA is composed of three parts; structural (C- E1 – E2), nonstructural (NS1 –NS2 - NS3 –NS4 - NS5), and two untranslated regions (5´-UTR and 3´-UTR) at two ends of the viral genome (1, 2). 5´-UTR region plays a major role in virion maturation, and nonstructural proteins have the main role in replication and polyprotein processing of the virus. Also 5´-UTR is a highly conserved region of the virus and is widely used to genotype the virus. This region has some specific internal ribosomal entry sites (IRES) in its structure which help to distinguish between genotypes and subtypes (3, 4). Heterogeneity in the virus is based on point mutations in 27 amino acids in this region, and the region is termed hyper variable region (HVR). Quasispecies mutations in HCV contribute to its survival against immune response; also, high genetic diversity with 13-18 mutations per year accounts for about 1.4-1.9 × 10-3 mutations per year within the entire genome (4, 5). Based on genetic differences among HCV isolates, hepatitis C virus species is classified into 6 genotypes with 120 subtypes (1-6).

HCV genotypes and subtypes are distributed worldwide affecting about 170 million people around the world. Genotypes 1-3 are common genotypes in the United States and Europe, genotype 5 is common in South Africa, genotype 4 is common in Egypt, and the common genotype in south Asia is 6 (7-9). The immigration in different geographical areas is the main reason for HCV genotypes distribution. Genotypes 1 and 3 are commonly found in Iran (10-12). Different HCV genotypes show different levels of resistance to antiviral drugs. Treatment regimens and patients response are different in various genotypes and subtypes of the virus. According to previous studies, infections with genotypes 2 and 3 have revealed better response to treatments than genotypes 1 and 4. Accordingly, HCV genotyping is more important for identification of HCV genotypes to prescribe the most effective regimen in appropriate dosage (13-15). Frequently HCV genotyping is performed by several different techniques: a. the golden standard analysis for HCV genotyping is direct sequencing of PCR products (16), b. Restriction fragment length polymorphism (RFLP) (17), c. Single strand conformation polymorphism (SSCP) and hetrodouplex analysis (18), d. using genotype specific primers (19, 20), f. Real-time PCR (21-24), g. DNA hybridization assay (25). Each of these methods has some advantages and disadvantages rendering it as an important issue to pursue further development of a more user friendly genotyping system.

## 2. Objectives

In this study we report the development of a homemade Line probe assay in Iran for the first time. Here, we developed a new diagnostic HCV genotyping method based on 5´-UTR and specific probes for each genotype followed by comparison with the results from RFLP genotyping assay.

## 3. Material and Methods

### 3.1. Serum Samples

A Total of 60 serum samples were collected from patients with positive results for HCV in Digestive Disease Research Center (DDRC) of Shariati Hospital, Tehran, Iran. All specimens had been tested for anti HCV antibody to be regarded as samples with positive results for HCV. To confirm the positive results of isolated serum samples regarding HCV, and to determine genotype of the isolates, RT-PCR RFLP had been previously used.

### 3.2. HCV RNA Extraction and cDNA Synthesis

50 μl of each sample was mixed with 450 μl of RNXTM (Cinnagen, Iran), and total RNA extraction was performed according to the manufacturer instructions. cDNA was synthesized with 0.1 μg of total RNA, using Revert Aid TM First Strand cDNA Synthesis Kit (Fermentas GmbH, Germany). Temperature and time conditions of the reaction were 25˚C for 5 min and 42˚C for 60 min and after cDNA synthesis, Reverse Transcriptase enzyme was inactivated at 75˚C for 5 min (26).

### 3.3. Nested PCR

5 μl of the each cDNA product was mixed with 20 μl of the PCR amplification solution, which contained 1× PCR buffer (Fermentas GmbH, Germany), 2 mM MgCl2, 0.2 mM dNTPs (Fermentas GmbH, Germany), 10 pmol/μl each outer primer (27) (M1 and M2) (Table 1), and 1 U of Taq DNA polymerase (Fermentas GmbH, Germany). The PCR was initiated at 94°C for 5 min, followed by 35 amplification cycles. The cycling protocol included denaturation at 94˚C for 1 min, followed by annealing at 55.5˚C for 1 min, and extension at 72°C for 1 min. A final extension was performed at 72°C for 5 min. A second PCR was performed with a set of inner biotinylated primers (27) (M3 and M4) (10 pmol/μl) (Metabion GmbH, Germany) using 1 μl of the first PCR product as DNA template. Thermal profile and components of the PCR tube were the same as the first PCR. Standard serum samples with positive and negative results were examined as controls in parallel with test samples. Detection of PCR products was performed through 2% agarose gel electrophoresis.

**Table 1.** Nucleotide Sequence, Position and Orientation of the Nested PCR Primers That Were Used to Amplify the 5’ UTR (23)

Primer name	Position	Polarity	Sequence^a^
**M1**	-299	+	5´- CCC TGT GAG GAA CTW CTG TCT TCA CGC-3´
**M2**	-1	_	5´- GCT CAT G R T GCA CGG TCT ACG AGA CCT -3´
**M3^b^**	-29	_	5´- CAC TCG CAA GCA CCC TAT CAG GCA GT-3´
**M4^b^**	-264	+	5´- TCT AGC CAT GGC GTT AGT A Y G AGT GT-3´

^a^Some of the primers contain degeneracy in nucleotide positions; W is A or T and R is A or G and Y is C or T

^b^The biotinylated inner primers

### 3.4. Preparation of the Line Probe Assay Strips

Two universal and seven specific probes for HCV type and subtypes were synthesized according to previous studies (Table 2) (25). The probes and the biotinylated inner primers (as positive control), were provided with the specific concentration (20 pmol /10 µl), and spotted onto SensiBlot^TM^ Plus nylon membranes (Fermentas GmbH, Germany) by using a 96-Well Dot-Slot Blotter (Cleaver, UK) according to the manufacturer instructions. The spotted membranes were fixed through baking at 80˚C for 30 min. Then the probes on the membranes were UV cross-linked with 100-150µJ/cm2. Finally the membranes were sliced into 7 mm strips width (4.2 cm2).

**Table 2.** Sequence, Position and Orientation of the Probes That Were Used to Genotype the HCV Isolates (21).

Probe name	Genotypes	Position	Sequence
**Uni1**	Universal1b 1c 6b 3a 3b 4a 2a 2c	-115	5´-TTG GGC GYG CCC CCG C-3´
**Uni2**	Universal1b 1c 6b 3a 3b 2a 2c 2b	-195	5´-TCT GCG GAA CCG GTG A-3´
**P3**	6a 6d 1a	-117	5´-TCT CCA GGC ATT GAG C-3´
**P4**	1b 1c 6b	-170	5´-AAT TGC CAG GAC GAC C-3´
**P5**	1b	-103	5´-CCG CGA GAC TGC TAG C-3´
**P12**	3a	-170	5´-AAT CGC TGG GGT GAC C-3´
**P13**	3a	-170	5´-AAT CGC TGG GGT GAC C-3´
**P14**	3a 3b	-103	5´-CCG CGA GAT CAC TAG C-3´
**P15**	3a 3b	-146	5´-TCT TGG AAC AAC CCG C-3´

### 3.5. Prehybridization, Hybridizations, and Color Development

Blocking solution is strongly recommended for prehybridization. A typical blocking solution containing 2X SSC [1X SSC: 0.15M NaCl (HiMedia, India) and 0.015 M Sodium Citrate (AppliChem GmbH, Germany)] and 0.1% SDD (Cinnagen, Iran) were added to each strip and incubated at 50˚C for 30 min. Hybridization was performed in the 14 ml polystyrene conical bottom test tubes. After adding a prehybridized stripe, 2 ml hybridization buffer (2X SSC 0.1% SDS) and 10μl denatured product, hybridization was performed at 50˚C for 1–2 hours with shaking at 80 rpm. The strips were washed twice with 2X SSC and 0.1% SDS for 5 min at room temperature and followed by washing twice with 1X SSC and 0.1% SDS for 15 min at 42˚C. After hybridization, developing of the stripes was performed by using Biotin Chromogenic detection kit (Fermentas GmbH, Germany) according to the manufacturer instructions. All stages were performed at room temperature on a platform shaker with gently shaking. Briefly, the strip was washed with 2ml Blocking/Washing buffer for 5 min, and then blocked in 2 ml of the Blocking solution for 30 min. Then the membrane was incubated in 2ml of diluted Streptavidin-AP conjugate for 30 min. After washing, to perform the enzymatic reaction and color development, the membrane was incubated in 2ml of freshly prepared Substrate solution [5-Bromo, 4-Choloro, 3-Indolylphosphate (BCIP) and Nitro-blue-tetrazolium (NBT)] in the dark. The blue-purple precipitate was visualized after 15-30 minutes of incubation. To stop the reaction, Substrate solution was discarded, and the membrane was rinsed with Milli-Q water for few seconds. Finally, the strips were interpreted by observing the created colored bands correspond to each genotype. The experiment was applied to all the 60 sera samples with positive results for HCV in triplicate, and diagnostic sensitivity and specificity comparison of the test regarding RT-PCR RFLP was computed. After analyzing the results by Fisher Exact Test, P-value between the two tests was calculated.

## 4. Results

**Figure 1.**
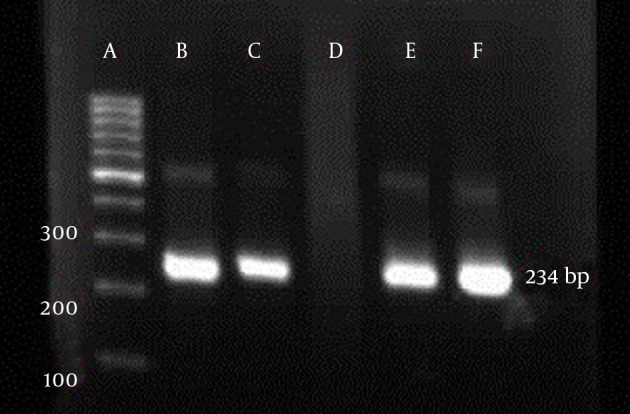
Nested PCR Amplification of 5’ UTR of the HCV Isolates. The 234 Band is the Biotinylated Inner Product. A: 100 bp DNA Ladder, B-C: Positive Control, D: Negative Control, E-F: Samples With Positive Results.

**Figure 2.**
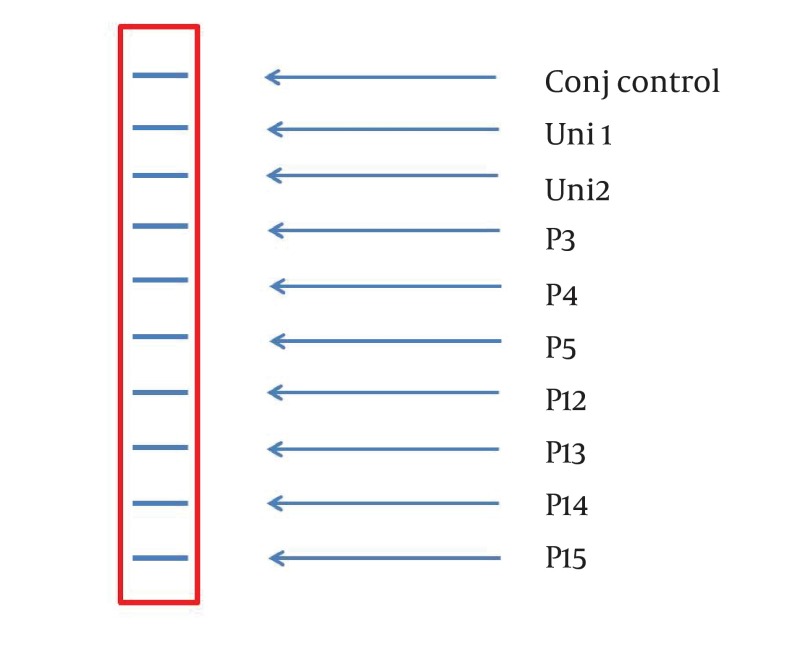
A Representative Stripe (6×0.7 cm) Containing all the Ten Parallel Probe Lines. Except Conj Control (Positive Control), Position of the Other Probes Is Corresponded to Information in Table 2.

**Figure 3.**
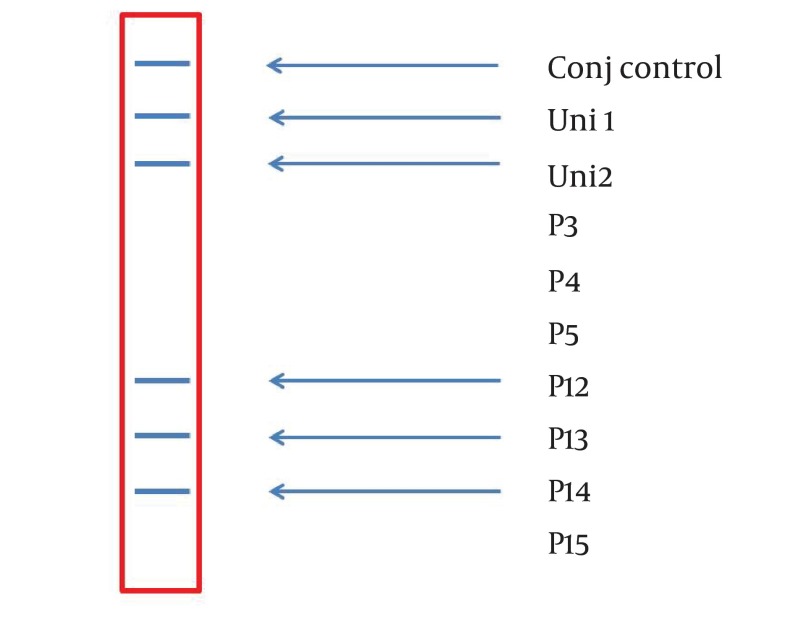
A strip Demonstrating the Presence of Genotype 3a Based on Reaction Among P12, P13, and P14 Specific Probes, and Corresponding Biotinylated Inner Product.

**Figure 4.**
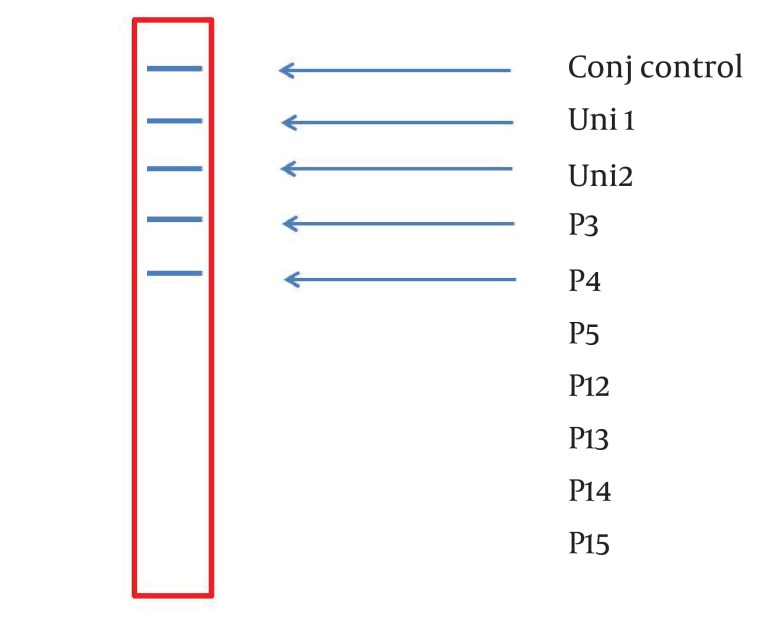
A Strip Demonstrating the Presence of Genotype 1a Based on Reaction Between P3 and P4 Specific Probes and Corresponding Biotinylated Inner Product.

**Figure 5.**
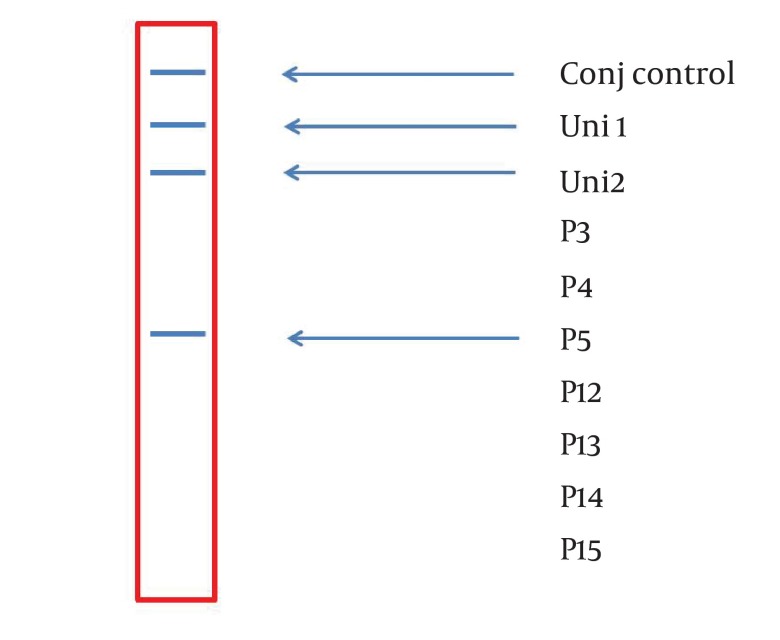
A Strip Demonstrating the Presence of Genotype 1b Based on Reaction Between P5 Specific Probe and Corresponding Biotinylated Inner Product.

**Figure 6.**
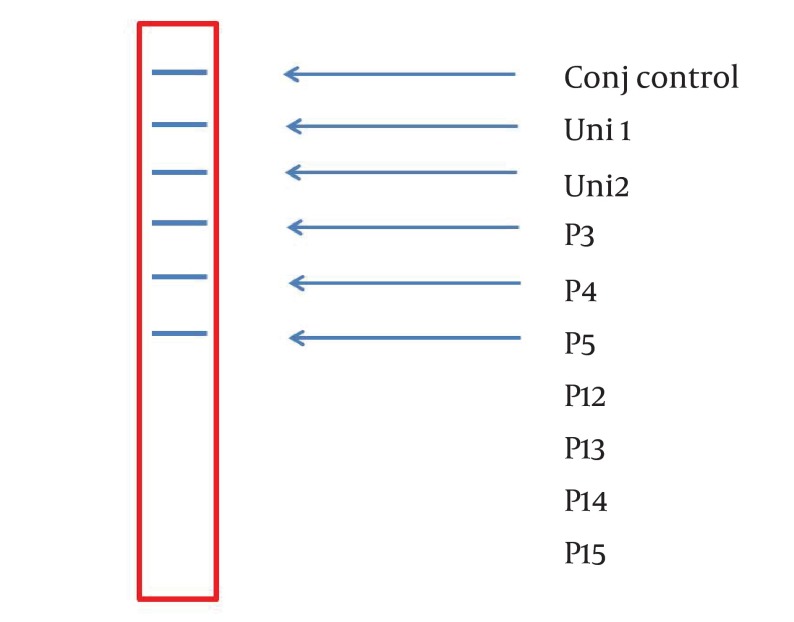
A Processed LiPA Strip Showing Simultaneous Presence of 1a and 1b Genotypes (Coinfection).

A 234 bp fragment from 5' UTR of HCV genome, was amplified using the two-step nested PCR. The initiation site of amplification was position of -299. The PCR products lengths were the same regardless of HCV genotype (Figure 1). Following hybridization of the biotinylated products to correspond spotted probes, biotin-streptavidin reaction was used to determine the genotypes. In this reaction 5-Boromo-4-Choloro-3-Indolyl Phosphate (BCIP) and Nitro-blue-tetrazolium (NBT) were used for colorimetric detection. During the reaction between BCIP/NBT and genotype, the detection was based on insoluble purple mixture production. The in-house LiPA was applied for discrimination of types 1and 3 in the first trial because they are so important in comparison with other types in Iran. It was our goal to detect Iranian epidemic genotypes 1a and 3 using seven separate immobilized probes on membrane while taking advantage of universal probes 1 and 2 that are highly conserved regions to confirm the PCR products. P3 and P4 probes directly determined HCV type 1, P5 probe was used for type 1b diagnosis, and P12 and P13 probes were used for genotype 3. In every strip, two universal probes were used for detection of all HCV genotypes and each strip had a conjugative control for confirmation of the reaction between biotin and streptavidin (Figure 2). It was concluded that 50% (n = 30) of HCV isolates of the 60 samples sera were belonged to 3a genotype (Figure 3), 18% (n = 11) to 1a genotype (Figure 4), 8% (n = 5) to 1b genotype (Figure 5), and 2% (n = 1) of sera were coinfected with 1a and 1b genotypes (Figure 6), while genotype of 22% (n = 13) of the sera was not identified by this method. Diagnostic sensitivity and specificity comparison regarding RFLP- PCR and the home made LiPA turned out to be 84% and 100% respectively. HCV genotype results were found to be in concordance through studying 60 sera that were successfully typed by the two methods. P-value of 0.045 conveys that the two methods were the same and had no significant difference. The tests performed in triplicate showed expected results confirming reproducibility of the assay. No need to agarose gel electrophoresis or using various genotype specific primers were the most important advantages of the home made LiPA. Also any point mutation in restriction enzyme site corresponding to a genotype may affect efficiency of PCR-RFLP while did not reduce the LiPA sensitivity.

## 5. Discussion

HCV genotyping is an epidemiological marker for prognosis of treatment response in patients. Therefore many efforts around the world have been devoted on HCV genotyping and detection. For achieving the mentioned goal, a simple, sensitive and reliable system is needed. HCV genotyping is performed by several different methods; although, sequence analysis is the gold standard for identification of HCV genotypes but is not practical for routine clinical laboratories (28). During the last 10 years, PCR based methods were developed which include: type-specific primers/probes (19) Restriction Fragments Length Polymorphism (17), melting curve analysis, and serological genotyping (29) hetrodouplex mobility analysis (18). In every single method only one region (NS5B, Core, and 5´-UTR) is analyzed. These methods have some advantages and disadvantages in HCV diagnosis of types and subtypes. This is the first time that HCV genotype determination has been performed by an in-house line probe assay in Iran. In our designed method, genotyping was performed and the results indicated that 50% of samples were belonged to 3a genotype, 18% 1a, and 8% 1b and 2% categorized in coinfection group. The most common subtypes in Iran are 1a and 3a, so we included their specific probes in our stripes (30-32). It is concluded that there is concordance between results of LIPA method and RFLP technique used for HCV genotyping. The most employed assays have been designed to identify genotypes and subtypes from the 5´-UTR of the HCV genome, because this region is highly conserved. However, 5´-UTR is still sufficiently variable to form type and subtype-specific motifs. These motifs can be detected and discriminated from each other by sequencing or reverse hybridization using specific probes (10, 19, 25). In this study we proposed an in-house method for diagnosis of HCV types and subtypes. It is important to note that simple, inexpensive and accurate method is needed; this study initiated to develop and design a reliable, sensitive, specific and economic genotyping assay for genotype determination of HCV samples in Iran. 

In conclusion, the LiPA permits rapid determination of the types and subtypes of HCV, and might aid detection of new HCV genotypes. Moreover this assay proved to be useful for further elucidation of the genotype-serotype relationships which is important to examine patients’ clinical status. This method provides possibility of evaluating more isolates without need for electrophoresis stage. Therefore the LiPA method can be a useful candidate for diagnostic purposes in Iran. Clearly, more studies using other methods such as direct sequencing are needed to validate the results. In this study we designed an in- house line prone assay genotyping method with high sensitivity, specificity and being capable of identifying HCV genotypes in Iran for the first time.
